# Smoking practices in relation to exhaled carbon monoxide in an occupational cohort

**DOI:** 10.1186/s12889-020-09997-4

**Published:** 2020-12-09

**Authors:** Denis Vinnikov, Zhangir Tulekov, Zhanna Romanova, Ilya Krugovykh, Paul D. Blanc

**Affiliations:** 1 Al-Farabi Kazakh National University, 71 Al-Farabi Avenue, Almaty, Kazakhstan 050040; 2 Peoples’ Friendship University of Russia (RUDN University), 6 Miklukho-Maklaya Street, Moscow, Russian Federation 117198; 3 National Research Tomsk State University, 36 Lenin Avenue, Tomsk, Russian Federation 634050; 4 University of California San Francisco, Suite 609, 350 Parnassus Avenue, San Francisco, CA 94117 USA

**Keywords:** Work-related, Carbon monoxide, Firefighting, Smoking, Waterpipe

## Abstract

**Background:**

Exposure to carbon monoxide (CO) remains a leading occupational hazard in firefighters, but cigarette and waterpipe smoking likely contributes to the other sources of CO in such workers. The aim of this study was to estimate the contribution of self-reported active cigarette smoking, waterpipe use, and potential job-related sources of CO to the level of exhaled CO in firefighters.

**Methods:**

We surveyed the personnel of 18 fire stations (*N* = 842), median age 28 years, who participated at an annual screening not timed to coincide with recent firefighting. We surveyed smoking and waterpipe history, exposure to secondhand smoke (SHS), use of coal for health and biomass for cooking and time since last exposure to firefighting in the workplace. We measured exhaled CO with an instantaneous reading device (piCO Smokerlyzer). We used multivariable regression models to test the association of time since last smoked cigarette (≤12 h) and waterpipe (≤12 h) and time since last fire (≤6 h) with exhaled CO.

**Results:**

In analysis limited to men (93.5% of all surveyed), 42% were daily cigarette; 1% were waterpipe smokers; 94% were exposed to SHS, 29% used coal for heating and 4% used biomass for cooking. The median CO was 4 (interquartile range 3;8) ppm. Age (beta 0.74 per 10 years, *p* < 0.001), use of biomass fuel for cooking (beta 1.38, *p* = 0.05), cigarette smoked in the last 12 h (beta 8.22, *p* < 0.001), waterpipe smoked in the last 12 h (beta 23.10, *p* < 0.001) were statistically associated with CO, but not time since last fire (≤6 h) (beta 4.12, *p* = 0.12). There was a significant interaction between older age and firefighting for exhaled CO (*p* = 0.03).

**Conclusions:**

Cigarette and recent waterpipe smoking are associated with increased exhaled CO in firefighters. Firefighting itself was a less potent contributor to exhaled CO when measured at an annual screening, but an age interaction was manifested.

**Supplementary Information:**

The online version contains supplementary material available at 10.1186/s12889-020-09997-4.

## Background

The occupational hazards of firefighting have been well characterized and prominently include exposure to high concentrations of carbon monoxide (CO), among a number of other combustion toxicants [1–3]. Similarly, exposure to combustion engine exhaust leads to CO inhalation such that professional drivers (including those in firefighting units) also may over-exposed occupationally. Cigarette smoking is a far more widespread source of CO exposure [4, 5] than either of these occupational sources and adds to the overall burden of CO in the workplace. Further, the emerging popularity of hookah (waterpipe) smoking, given its particular association with CO exposure, adds another potential contributor to CO in the occupational context [6]. In the current analysis, we estimated the contribution of self-reported active cigarette smoking, waterpipe use, and potential job-related sources of CO to the level of exhaled CO in firefighters.

## Methods

### Study design and questionnaire

We enrolled the staff of all 18 rescue and firefighting departments located in Almaty, Kazakhstan. Recruitment took place during the employees’ annual mandated work-based surveillance screening. All of the workers undergoing annual surveillance who were screened before the program’s suspension due to COVID-19 (in March 2020) were eligible for inclusion in the analysis (*N* = 842).

Participants completed questionnaires eliciting current occupational position, work duration, age, sex, direct cigarette smoking, waterpipe smoking, regular exposure to secondhand smoke, use of biomass fuel for cooking and coal for heating, and place of residence (urban vs. rural). The questionnaire was developed for this study (see Additional file 1). For those in active firefighting jobs (firefighter, senior firefighter, shift commander, division head, department head and firetruck driver), we elicited hours elapsed since last present at the scene of a fire. Self-reported smoking status was categorized as never-smoker, ex-smoker, or current smoker. The number of smoked cigarettes per day was elicited in current smokers and smoking duration in years elicited in current and ex-smokers. For current cigarette and waterpipe smoking, the number of hours elapsed since last use was asked at the time of surveillance examination.

### Exhaled CO measurement

In all participants we measured exhaled CO using an instantaneous reading CO monitor (Smokerlyzer, Bedfont, UK). Levels in ppm were rounded to the nearest whole integer. For CO measurement, subjects were asked to hold their breath for 15 s and then exhale smoothly into the disposable mouthpiece of the monitor. The device is calibrated annually. The producer reports ±2 ppm (5%) accuracy and a range 0 to 150 ppm. Exhaled CO measurement was also accompanied by a brief counseling intervention with details on the typical sources of CO and along with advice to cease cigarette and waterpipe smoking as applicable.

### Statistical analysis

Because after cessation of environmental exposure, the concentration of exhaled CO falls off with a half-life of 6 h [7], we hypothesized that time since exposure to suspect sources would predict the concentration of exhaled CO. To assess this, we created two time-based variables each for firefighting, cigarette smoking, and waterpipe use. For firefighting, we created one variable for exposure that occurred <=6 h prior to CO measurement (e.g., one half-life) and a separate variable for current firefighting but not within the last 6 h. Because no waterpipe smokers reported use within 6 h, we defined one variable as with 12 h and second as current waterpipe use but not within 12 h of testing (e.g., two half-lives). We used a parallel approach to define variables for current cigarettes smoking.

We used the Kruskall-Wallis test or the chi square for comparisons among seven occupational groups. From the overall sample *N* = 842, only 6.5% were female (Table 1). Participant work and smoking characteristics likely to be associated with CO, as well as potential confounding variables, differed systematically by gender. Consistent with those differences, the median exhaled CO among the females was half that of the men (median 2.0 ppm vs. 4.0 ppm, *p* < 0.01). Based on this preliminary analysis, we therefore excluded the female subset of employees from further analysis, limiting the study group to men only (*N* = 787).

**Table 1:** Demographic, lifestyle and exhaled CO profile of included subjects

Subject Characteristics	Overall	Men	Women	p
All subjects, N (%)	842 (100)	787 (93.5)	55 (6.5)	–
Age, years, median (IQR)	28 (25;36)	28 (25;36)	36 (28;40)	< 0.001
Height, cm, median (IQR)	175 (170;179)	175 (171;179)	164 (161;169)	< 0.001
Urban residence, N (%)	429 (51)	389 (49)	40 (73)	< 0.001
Work duration, years, median (IQR)	6 (3;14)	5 (3;13)	12 (5;18)	< 0.001
Years in first response, median (IQR)	3 (1;7.25)	3 (1;8)	0 (0;0)	< 0.001
Smoking status				
Never smokers, N (%)	282 (34)	247 (31)	35 (64)	< 0.001
Ex-smokers, N (%)	222 (26)	210 (27)	12 (22)	
Current smokers, N (%)	338 (40)	330 (42)	8 (14)	
Waterpipe smokers, N (%)	11 (1)	11 (1)	0 (0)	0.38
Exposed to SHS, N (%)	783 (93)	738 (94)	45 (82)	< 0.001
Fossil fuel users for heating, N (%)	236 (28)	232 (29)	4 (7)	< 0.001
Biomass fuel users for cooking, N (%)	34 (4)	34 (4)	0 (0)	0.12
Walking 6 km daily, N (%)	458 (54)	437 (52)	21 (38)	< 0.05
Exercising 3 times a week, N (%)	538 (64)	523 (66)	15 (27)	< 0.001
Exhaled CO, ppm, median (IQR)	4 (2;8)	4 (3;8)	2 (2;3)	< 0.001

*IQR* Interquartile range Pairwise differences in characteristics were tested using non-parametric methods: the Mann-Whitney U-test for continuous variables or the chi-square test for categorical variables

Using bivariate analyses among the male participants, we tested whether exhaled CO was associated with recent exposure (≤ 12 h since last waterpipe use, ≤ 12 h since last cigarette smoked, ≤ 6 h since last firefighting event), regular exposure to secondhand smoke (SHS), use of biomass fossil fuel cooking, use of coal for home heating, urban vs. rural residence, height, and age. Because duration of employment was strongly correlated with age (*r* = 0.92), we did not test the former as an independent predictor variable for exhaled CO. We also tested multicollinearity of all predictor variables and found variance inflation factor for all variables below 10, indicative of no evidence of it. In a correlation matrix we found that urban residence and the use of biomass for heating were moderately correlated (*r* = − 0.46), whereas use of biomass for heating and cooking had some weak correlation (*r* = 0.29). We used multivariable linear regression modelling to test the associations between selected independent predictors and exhaled CO, including variables from the bivariate analysis that achieve a cutoff of *p* ≤ 0.20. In such multivariate regression analysis, regression coefficients of the variables of interest reflect adjustment for all included predictors, in the model we present, age, urban residence, exposure to SHS, use of coal for heating and biomass for cooking, as well as three time-related smoking, waterpipe smoking, and firefighting variables. We report the parameter estimate for each predictor tested with its corresponding *p*-value. In additional analyses, we also re-estimated the multivariable linear regression model stratifying at the median age of the cohort (28 years) in order to assess the potential effect modification of age. We further tested interaction terms for age where initial analysis suggested effect modification. We ran all analyses in NCSS 2019 (Utah, USA). The study was approved by the Committee on Bioethics of al-Farabi Kazakh National University.

## Results

We grouped the 787 male employees into seven occupational categories based on their current position (Table 2). This included 329 active firefighters (median age 26 years, interquartile range [IQR] 24 to 28 years). Those employees served in positions of either a firefighter or a senior firefighter. There were also 169 firetruck drivers and driving instructors in the cohort (median age 30 years, IQR 25 to 37.5 years); 148 shift commanders and division heads (median age 36 years, IQR 30 to 40.75 years); 31 department heads and assistant chiefs (median age 34 years, IQR 28 to 41 years); a group of 16 foremen and mechanics (median age 30.5 years, IQR 27 to 35.25 years); 56 engineers and senior engineers (median age 28.5 years, IQR 26 to 34 years); and, finally, 38 others with a variety of jobs not otherwise categorized (median age 33.5 years, IQR 27.75 to 39.25 years). Firefighters (of all positions), shift commanders, division heads, department heads with their assistant chiefs, along with professional drivers are the job classifications with duties that include attending the scene of a fire or potentially being exposed to combustion fumes or engine exhaust. The remaining personnel typically work in the equipment garage or in administrative offices, without fire or combustion engine sources of CO exposure.

**Table 2:** Occupational status, smoking status, waterpipe use, and exhaled CO of the cohort

Occupational Category	Frequency N (%)	Age in years, median (IQR)	Current cigarette smokers N (%)	Waterpipe smokers N (%)	Exhaled CO in ppm, Median (IQR)
All Occupational Groups	787 (100)	28 (11)	330 (42)	11 (1)	4 (5)
1. Firefighters	329 (42)	26 (4)	114 (35)	9 (3)	4 (5)
2. Firetruck drivers and driving instructors	169 (21)	30 (12.5)	80 (47)	0 (0)	5 (7)
3. Shift commanders and division heads	148 (19)	36 (10.8)	74 (50)	1 (1)	4 (7)
4. Department heads or assistant heads	31 (4)	34 (13)	14 (45)	0 (0)	4 (6)
5. Senior and junior foremen and respiratory equipment mechanics	16 (2)	30.5 (8.3)	12 (75)	0 (0)	6 (8)
6. Senior or junior engineers	56 (7)	28.5 (8)	21 (38)	1 (2)	3.5 (6.8)
7. Others	38 (5)	33.5 (11.5)	15 (39)	0 (0)	3 (6.3)

*CO* carbon monoxide, *IQR* interquartile range

Exhaled CO level equaled 3 (IQR 2) ppm in self-reported cigarette never-smokers; 3 (IQR 2) ppm in self-reported ex-smokers and 10 (IQR 8) ppm in current smokers.

The largest of seven occupational groups was firefighters (Table 2), while group of foremen and mechanics was the smallest, comprising only 2% of study group. The median exhaled CO for the group overall was 4 ppm (IQR 3 to 8) with significant differences among the groups (Kruskall-Wallis test *p* < 0.001). The highest median value was among foreman and mechanics group and the next highest among fire truck drivers and instructors. Firefighters, however, manifested a median CO value matching that of the group as a whole. There were significant differences in age among the seven groups (Kruskall-Wallis *p* < 0.001) with more employees of younger age working as firefighters, and older population falling under the manager/department head group. There were 42% current smokers in the entire male group, with the greatest proportion in foreman and equipment group (75%) and lowest among firefighters (35%). The variation in smoking prevalence among the seven groups also was statistically significant (*p* < 0.01). The Spearman correlation between proportion of current smokers and the median exhaled CO for each group was 0.67, but did not achieve statistical significance (*p* = 0.11). Current smoking intensity was less than one pack per day (median = 7 cigarettes; IQR 4 to 10), with an average duration under 10 years (median 7; IQR 4 to 14.25 years). Only 1 % of study participants reported current waterpipe use. Waterpipe smokers were confined to only three of the seven occupational groups (Table 2).

In bivariate analyses, exhaled CO was statistically greater among those who were older, smoked a cigarette in the last 12 h, used a waterpipe in the last 12 h, were exposed to SHS at work or at home, lived in a rural area outside the city, use of coal for heating and use of biomass to cook (Table 3). We observed the strongest effect from recent waterpipe use (beta 19.7), a moderate effect from recent cigarette smoking (beta 8.5), and smaller estimated effects from exposure to SHS, use of coal for heating, biomass for cooking, and from being a rural resident. Being at the scene of a fire in the last 6 h was associated with a moderate effect (beta 5.2) but this did not achieve statistical significance.

**Table 3:** Subject characteristics associated with exhaled CO and their predictive values in unadjusted and fully adjusted regression analyses

Predictor	Bivariate Analyses			Multivariate Analysis	
	R^2^	beta	P	beta	P
Age (per 10 years)	0.06	2.07	< 0.001	0.74	< 0.001
Height	0.0008	−0.03	0.43	NA	NA
Urban residence	0.02	−1.56	< 0.001	−0.20	0.52
Exposure to SHS	0.02	2.85	< 0.001	0.49	0.39
Coal use for heating	0.02	1.57	< 0.001	0.36	0.30
Biomass fuel use for cooking	0.004	1.72	0.09	1.38	0.05
Last waterpipe use ≤12 h	0.03	19.72	< 0.001	23.10	< 0.001
Last cigarette ≤12 h	0.50	8.49	< 0.001	8.22	< 0.001
Last fire ≤6 h	0.002	5.19	0.20	4.12	0.12

*NA* not applicable, *SHS* secondhand smoke

In the multivariate analysis, the overall *R*^*2*^ = 0.56

In multivariable analysis, including age, urban residence, exposure to SHS, use of coal for heating and biomass for cooking, as well as three time-related smoking, waterpipe smoking, and firefighting variables, the strongest predictors of exhaled CO were recent cigarette smoking and recent waterpipe use (Table 3). All 787 subjects were included in this multivariable analysis. Recent waterpipe use increased exhaled CO by 23 ppm, whereas recent cigarette smoking by 8.5 ppm. In the same analysis, being at the scene of fire was not significantly associated with a CO increase (*p* = 012). The overall model R^2^ was 0.56.

**Fig. 1:**
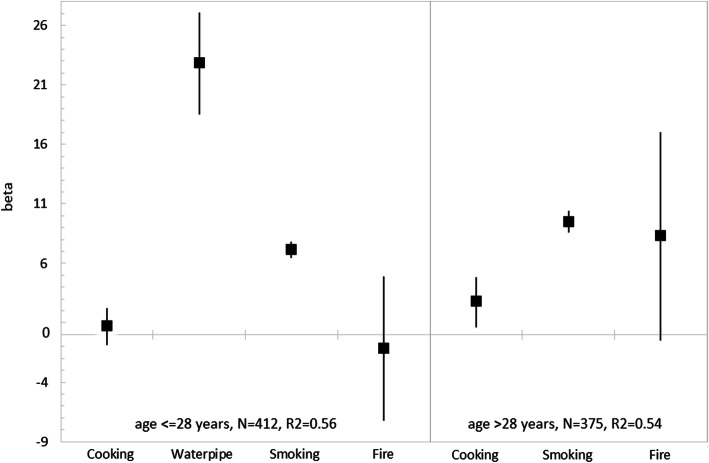
Betas and their corresponding 95% CIs for selected predictors in the multivariate regression models in those below or median age (on the left) and above median age (on the right). There were 48 employees of the median age (28 years), and they were included in the first group

Limiting the predictive model to biomass fuel use, recent smoking, recent waterpipe use, and recent firefighting, we repeated the multivariate analysis stratified by the median age of the entire cohort (28 years) (Fig. 1). Recent waterpipe use could not be studied in older workers because there were no such users in that stratum. The beta estimate for smoking was similar in younger compared to older workers (beta 7.12 vs 9.45, *p* < 0.001 for both) (Fig. 1). The association of cooking with biomass fuel with CO was present in the older stratum (beta 2.73, *p* < 0.01), but was negligible in the younger stratum (beta 0.65, *p* = 0.41). The largest potential effect modification by age was evident for being on the scene of a fire within 6 h: beta 8.27 (*p* = 0.06) among older workers compared to beta − 1.18 (*p* = 0.71) in the younger stratum. Further analysis testing the association of exhaled CO with age, firefighting within 6 h, and an age*firefighting interaction term, there was a statistically significant interaction (*p* = 0.03).

## Discussion

In this study of close to 900 firefighters and allied occupations, all working in the fire department of Almaty, the largest city in Kazakhstan, we ascertained the contribution to exhaled CO of cigarette and waterpipe smoking, exposure to recent active firefighting, use at home of coal for heating and biomass fuel for cooking. Given the biological half-life of CO and thus the critical role of time since exposure in measured CO, we based our analysis predicated on time elapsed since smoking cigarettes or a waterpipe and being recently at the scene of fire. We found that recent waterpipe and cigarette smoking are strongly related to exhaled CO. Although waterpipe smoking was less common than cigarette smoking, the former had a greater effect on exhaled CO. Recent exposure to a fire was associated with CO only among older fighters.

Traditional charcoal-fired waterpipe smoking, which in the last 10 to 15 years has become more popular in Kazakhstan predominantly among younger persons, is associated with higher levels of CO compared to other ways of smoking, such as smoking conventional cigarettes or even electrically heated hookahs [8]. With similar concentrations of saliva cotinine reflecting similar nicotine delivery in cigarette and waterpipe smokers, the levels of CO measured through biomonitoring differ dramatically between these two popular ways of smoking [9]. Moreover, a meta-analysis of studies comparing toxicant levels from cigarette and waterpipe smoking showed that in addition to higher pooled CO (nearly 10-fold higher for waterpipe smoking compared to a single cigarette), waterpipe smokers were exposed to much greater volumes of smoke itself (74.1 vs. 0.6 l) [10].

Overexposure to CO has been assessed as an occupational issue among the employees of hookah cafes [11–15]. Not surprisingly, these studies have shown, through atmospheric and biomonitoring, that CO exposure is elevated. Elevations in other indoor air pollutants have also been documented, including black carbon and PM_2.5_ [14]. In one study [11], CO exposure concentrations exceeded occupational exposure guidelines, putting the personnel at increased risk of adverse health effects associated with CO intoxication [11]. Even in a study reporting relatively low exhaled CO concentrations in the employees, the number of waterpipe smokers in the venue studied was positively associated with exhaled CO in the staff [15]. Even among non-smoking employees, exhaled CO levels may reach 27 ppm in the waterpipe bars [12]. Despite these findings, hookah-associated CO over-exposure from personal use in workers other than those directly employed by hookah bars has not been considered from an occupational perspective, especially among persons already at risk from work-related CO. Our findings suggest that this factor should indeed be taken into account in occupational environments with traditional CO exposures, along with other sources that, like waterpipe use, may vary depending on local factors.

Firefighters are one such occupation because CO is considered a major potential on-the-job exposure, along with a wide range of other inhalants that also are released through combustion. Elevations in CO have been documented in biomonitoring among both urban and wildland firefighters [1, 3, 16]. Furthermore, this has been observed despite reported compliance with personal protective equipment use requirements [17]. Because work-related CO exposure risk can be mitigated but not necessarily eliminated among firefighters, taking into account concomitant sources of exposure in this group can be particularly relevant to occupational health management for such workers. Our data suggest this may be even more relevant among older firefighters.

Almaty is a city with high reported levels of air pollution, whereas the exposure to combustion products from fossil fuel use for heating and cooking from the suburb and heating plants in winter may be high [18]. Ambient air pollution may result in higher exhaled CO levels in those living in highly polluted cities [19]; however, the contribution of ambient CO in our cohort was negligible compared to cigarette smoking and waterpipe use because among 452 non-current smokers and never-waterpipe users, 180 (40%) had exhaled CO 0, 1 or 2 ppm.

The strengths of this analysis should be noted. First, ours is a novel observation of exhaled CO in relation to time from the occupational exposure to firefighting, cigarette smoking and waterpipe smoking. These factors accounted for more than 50% of the variability in CO in our occupational cohort of firefighters. Secondly, we also considered important confounders of the association often missed in other studies, including the use of coal at home for heating and biomass fuel for cooking. Importantly, by studying firefighters in a major Central Asian city, we contribute observations from a geographic region rarely studied from an occupational environmental health perspective. Finally, uniform data collection at the annual screening and large sample size yielded consistency across fighters from various locations in the city across a range of work tasks and job assignments. The limitations of our study include the inability to measure systematically exhaled CO immediately after return to fire station after a firefighting event, which would better have explained variability in CO in our cohort. Since all fighters were examined at their annual screening, very few of them had short time since exposure, because many are referred to screening at the end of vacation or multiple days off work. Furthermore, the outbreak of COVID-19 entailed abrupt screening cessation, reducing the overall sample size. Another limitation is predominance of males in this cohort, potentially lessening the contribution of exposure to biomass fuel for cooking, because traditionally women may be more likely to be exposed to fire while cooking. Finally, we could not consider all potential environmental and occupational sources of CO; therefore, some unmeasured confounding in this analysis may be present.

## Conclusions

Individual waterpipe and cigarette smoking, and to a lesser extent exposure to CO at the scene of fire, were the leading factors associated with elevated CO in a cohort of firefighters. Concomitant sources of CO exposure in this group can be particularly relevant to occupational health management for such workers, because elimination of exposure to CO at the scene of fire is not always possible and many of the job task in a firefighter’s cohort such as ours do not entail active firefighting duties. Cigarette and waterpipe smoking cessation should be recommended to reduce the individual exposure to CO in these occupations.

## Supplementary Information

**Additional file 1:.** Study questionnaire. The English version of the questionnaire in the study

## Data Availability

The datasets used and/or analysed during the current study are available from the corresponding author on reasonable request.

## Abbreviations

CI: Confidence interval
CO: Carbon monoxide
IQR: Interquartile range
Ppm: Parts per million
SHS: Secondhand smoke
