# Supplementary material for: Smoking practices in relation to exhaled carbon monoxide in an occupational cohort
Source: BMC Public Health. 2020 Dec 9;20:1894. doi: 10.1186/s12889-020-09997-4 (PMC7725030; doi:10.1186/s12889-020-09997-4)
Supplement: Supplementary file 1 — Additional file 1:. Study questionnaire. The English version of the questionnaire in the study [file 12889_2020_9997_MOESM1_ESM.pdf]

PARTICIPANT’S QUESTIONNAIRE

1. Full name ____
2. Mobile phone ____________________________
3. Date of birth (DD/MM/YYYY) ________________
4. Sex: Male Female
5. Address ____
6. Your overall work duration: ________ full years
7. Provide details about your current position:

Position	Work shift duration	Number of shifts per week	Work duration (years)

8. Please list your last four positions including the one you work on now:

Position	Years

9. Do you use respiratory personal protective equipment (respirator, gasmask, etc.)?
   □ No (tick and go to question 11)
   □ Yes
10. How often do you use respiratory personal protective equipment (respirator, gasmask, etc.)?
   □ Seldom
   □ Almost half of the work time
   □ Most of the work time
   □ Always
11. Have you ever smoked cigarettes?
   □ No (tick and go to question 16)
   □ Yes
12. Do you continue to smoke at present?
   □ No (tick and go to question 16)
   □ Yes
13. Do you smoke daily?
   □ No (tick and go to question 16)
   □ Yes
14. How many cigarettes a day do you smoke? _______
15. What is your smoking duration in years? _________
16. Do other people smoke near you at home or in the workplace?
   □ No
   □ Yes

16a. Do you smoke waterpipe at present?
   □ No
   □ Yes

17. Do you use fossil or biomass fuel (NOT NATURAL GAS OR PROPANE) for heating at home?
   □ No
   □ Yes
18. Do you use fossil or biomass fuel (NOT NATURAL GAS OR PROPANE) for cooking at home?
   □ No
   □ Yes
19. Do you walk 6 km or make 10 thousand steps a day, including weekends?
   □ No
   □ Yes
20. Are you involved in any regular physical activity at least 3 times a week?
   □ No
   □ Yes
21. Please choose one option of four listed below which best describes your alcohol consumption:
   □ I never drink
   □ I drink seldom (less than once a week) in small amounts

□ I drink seldom (less than once a week) in moderate amounts
□ I drink at least once a week

22. The following questions relate to your respiratory symptoms (CAT questionnaire). For each item below, place a mark (X) in a box that best describes you currently. Be sure to only select one response for each question

A	I never cough	0 1 2 3 4 5	I cough all the time
B	I have no phlegm (mucus) in my chest at all	0 1 2 3 4 5	My chest of completely full of phlegm (mucus)
C	My chest does not feel tight at all	0 1 2 3 4 5	My chest feels very tight
D	When I walk up a hill or one flight of stairs I am not breathless	0 1 2 3 4 5	When I walk up a hill or one flight of stairs I am very breathless
E	I am not limited doing any activities at home	0 1 2 3 4 5	I am very limited doing activities at home
F	I am confident leaving my home despite my lung condition	0 1 2 3 4 5	I am not at all confident leaving my home because of my lung condition
G	I sleep soundly	0 1 2 3 4 5	I don't' sleep soundly because of my lung condition
H	I have lots of energy	0 1 2 3 4 5	I have no energy at all

23. This question is about your breathlessness (mMRC questionnaire). Please choose only one answer that applies to you:

**Question**	**Score**
I only get breathless with strenuous exercise	0
I get short of breath when hurrying on a level or walking up a slight hill	1
I walk slower than people of the same age on the level because of breathlessness, or a I have to stop for breath when walking on my own pace on the level	2
I stop for breath after walking about 100 meters of after a few minutes on the level	3
I am too breathless to leave the house of I am breathless when dressing or undressing	4

24. Have you ever had wheezing in your chest (especially when you exhale)?
□ No (tick and go to question 26)
□ Yes

25. Have you ever woken up at night or on early morning because of wheezing in your chest?
□ No
□ Yes

26. Compared to the people of your age, are you more breathless?
□ No
□ Yes

27. Do you have seasonal allergy (runny nose, nasal congestion in spring, summer or early fall)?
□ No
□ Yes

28. Has the doctor ever told you that you have chronic bronchitis?
□ No
□ Yes

29. Has the doctor ever told you that you have chronic obstructive pulmonary disease?
□ No
□ Yes

30. Has the doctor ever told you that you have bronchial asthma?
□ No
□ Yes

31. Has the doctor ever told you that you have allergic rhinitis
□ No
□ Yes

32. How many hours have elapsed since your last waterpipe smoking?

33. How many hours have elapsed since your last cigarette smoking? ________________________

34. How many hours have elapsed since your last presence at the scene of fire? ___________________
